# Genome Sequences of Two Dual-Serotype-Specific Echovirus 20 Strains from Nigeria

**DOI:** 10.1128/MRA.00894-19

**Published:** 2019-10-24

**Authors:** T. O. C. Faleye, O. M. Adewumi, D. Klapsa, M. Majumdar, J. Martin, J. A. Adeniji

**Affiliations:** a Department of Virology, Faculty of Basic Medical Sciences, College of Medicine, University of Ibadan, Ibadan, Nigeria; b Centre for Human Virology and Genomics, Department of Microbiology, Nigerian Institute for Medical Research, Lagos, Nigeria; c Infectious Diseases Institute, College of Medicine, University of Ibadan, Ibadan, Nigeria; d Division of Virology, National Institute for Biological Standards and Control, Potters Bar, Hertfordshire, United Kingdom; e WHO National Polio Laboratory, University of Ibadan, Ibadan, Nigeria; Portland State University

## Abstract

Here, we describe nearly complete genome sequences (7,361 nucleotides [nt] and 6,893 nt) of two echovirus 20 (E20) isolates from Nigeria that were simultaneously typed as CVB and E20 (dual serotype) by neutralization assay. Both include two overlapping open reading frames (ORFs) of 67 and 2,183 amino acids that encoded a recently described gut infection-facilitating protein and the classic enterovirus proteins, respectively.

## ANNOUNCEMENT

Enteroviruses (EVs) are members of the genus Enterovirus, family Picornaviridae, and order Picornavirales. Until the early 2000s, nonpolio enterovirus (NPEV) identification was by neutralization using polyclonal antisera (1). However, with correlation established between EV serological types and sequence data of the VP1 region (2), VP1 amplification and sequencing (molecular identification) became the gold standard for identification (3). One of the biological phenomena yet to be accounted for by molecular identification is dual-serotype specificity (DSS) (4, 5), a situation in which the neutralization typing assay identifies an isolate as simultaneously belonging to two different serotypes. Here, we describe the genomes of two echovirus 20 (E20) isolates from Nigeria that were simultaneously typed as coxsackievirus B (CVB) and E20 (dual-serotype specific) by the Netherlands National Institute for Public Health and the Environment (Rijksinstituut voor Volksgezondheid en Milieu [RIVM]) enterovirus typing antisera (1).

Two E20 strains isolated in Nigeria are described here. E20_2010 was recovered from sewage-contaminated water in 2010 (6). E20_2014 was recovered from the stool of a child with acute flaccid paralysis (AFP) in 2014 (our unpublished data). Both strains were isolated on a human rhabdomyosarcoma cell line (1) and subjected to a neutralization assay (1) and molecular identification (3). Both isolates were further subjected to RNA extraction, cDNA synthesis, and full-genome amplification by sequence-independent, single-primer amplification (SISPA) (7, 8) for isolate E20_2010 or to reverse transcription-PCR (RT-PCR) of overlapping fragments for isolate E20_2014 (9), followed by Illumina sequencing.

E20_2010 library preparation was done using the Nextera XT DNA sample preparation kit, and paired-end sequencing (2 × 301 bp) for 300 cycles was done using the MiSeq system (Illumina). Trimming and assembly were done using Geneious R10 software (7). E20_2014 library preparation was done using the Nextera DNA sample preparation kit. Paired-end sequencing (2 × 150 bp) for 300 cycles was done using the HiSeq system (Illumina). Trimming and assembly were done using Trimmomatic (v1.2.14) and Kiki (v0.0.9), respectively. All software was used with default settings.

The genome of E20_2010 contains 7,361 nucleotides (nt) (positions 24 to 7384 relative to those of the JV-1 E20 prototype strain [GenBank accession number AY302546; similarity = 77.25%]) assembled from 101,921 (23.68%) of the 430,320 filtered reads generated. It has a G+C content of 48%. The two overlapping open reading frames (ORFs) (10) have 67 and 2,183 amino acids (aa), respectively.

For the E20_2014 genome, two nonoverlapping contigs were assembled from 1,103,508 (29.44%) of the 3,748,102 reads generated. The missing region was recovered by RT-PCR with specific primers and Sanger sequencing. The final assembly has 6,893 nt (positions 497 to 7391 relative to those of the JV-1 E20 prototype strain [similarity = 76.64%]). The two overlapping ORFs and G+C content are exactly as in E20_2010. The Molecular Evolutionary Genetics Analysis 5 (MEGA5) pairwise distance estimator (using the Kimura 2-parameter model) showed that both genomes are 81.65%, 88.13%, and 81.40% similar at the nucleotide level for the genome, ORF1, and ORF2, respectively. They are, however, 82.07% and 96.36% similar at the amino acid level for ORF1 and ORF2, respectively. These genomes will now serve as references for delineating the biological basis of dual-serotype specificity.

### Data availability.

The assembled genomes have been deposited in GenBank under the accession numbers MN181513 and MN181514. The raw reads have been deposited in SRA (accession numbers SRX6451376 and SRX6451375 for E20_2010 and E20_2014, respectively) under the project accession number PRJNA554815.
